# AI in humanitarian healthcare: a game changer for crisis response

**DOI:** 10.3389/frai.2025.1627773

**Published:** 2025-07-02

**Authors:** Diala Haykal, Mohamad Goldust, Hugues Cartier, Patrick Treacy

**Affiliations:** ^1^Centre Laser Palaiseau, Palaiseau, France; ^2^Department of Dermatology, Yale University School of Medicine, New Haven, CT, United States; ^3^Centre Médical Saint Jean, Arras, France; ^4^Ailesbury Clinics Ltd., Dublin, Ireland

**Keywords:** artificial intelligence, humanitarian healthcare, crisis response, disaster management, ethical considerations

## Abstract

Artificial Intelligence (AI) is transforming humanitarian healthcare by providing innovative solutions to critical challenges in crisis response. This review explores peer-reviewed literature and case reports from 2001 to 2025, retrieved from PubMed, Scopus, and Google Scholar, using targeted keywords. Results indicate that AI enhances disaster prediction, disease surveillance, resource allocation, and mental health support through tools such as machine learning, natural language processing, robotics, and blockchain. Prominent applications include AI-powered early warning systems, chatbots for displaced populations, telemedicine platforms, and automated supply chain logistics. Ethical concerns such as data privacy, bias, and access inequities remain critical to responsible deployment. By uniting governments, NGOs, and technology providers, AI serves as a powerful tool to strengthen humanitarian healthcare systems, enhancing resilience and efficiency while ensuring better outcomes for vulnerable populations during crises.

## Introduction

Artificial Intelligence (AI) is revolutionizing humanitarian healthcare by offering innovative solutions to some of the most complex challenges faced in crisis response (Alowais et al., 2023). From managing natural disasters to aiding displaced populations, AI-driven technologies are enhancing efficiency, improving medical interventions, and enabling faster decision-making in emergency situations (Ghaffarian et al., 2023; Calle Müller et al., 2024; Bari et al., 2023). The ability of AI to predict disasters, optimize resource allocation, and facilitate real-time communication makes it an invaluable tool for humanitarian efforts (Abdul et al., 2024). As global crises become increasingly unpredictable, the integration of AI into healthcare initiatives is transforming the way aid is delivered, ensuring that vulnerable populations receive timely and effective assistance (Albahri et al., 2024).

## Methodology

This review is based on a targeted literature search covering the period from 2010 to February 2025. We systematically searched databases including PubMed, Scopus, and Google Scholar using combinations of keywords such as “artificial intelligence,” “AI in crisis response,” “humanitarian healthcare,” “AI in disaster management,” and “AI refugee health.” Articles were included if they described the development, application, or evaluation of AI technologies in humanitarian or crisis-related healthcare contexts. Both peer-reviewed articles and high-impact case studies were prioritized. Table 1 summarizes key tools and initiatives identified repeatedly across the literature or noted for their real-world impact.

**Table 1:** AI applications in humanitarian healthcare.

Application name	Purpose/description	Ethical considerations
CIMA (Children immunization App)	Boosts vaccination adherence among refugee children	Informed consent, data privacy of children
Wysa	AI-powered mental health chatbot using CBT and mindfulness	Confidentiality, lack of human oversight
Woebot	AI chatbot for psychological support	Data protection, appropriateness in acute mental health crises
IBM Watson Health	Supports disease tracking and intervention (e.g., malaria control)	Bias in data sets, transparency of decision-making
ZzappMalaria	AI-powered app for identifying mosquito breeding sites to combat malaria	Location data sensitivity, equitable deployment
Google BERT	NLP tool for real-time translation in crisis communication	Risk of mistranslation, bias in language models
OpenAI Models	Used for NLP to enhance health communication and emergency alerts	Misinformation control, contextual sensitivity
MERON (by Kimetrica)	AI tool for rapid image-based malnutrition screening	Biometric data use, consent in vulnerable populations
Google Flood Forecasting Initiative	Predicts floods using real-time data and AI algorithms	Accuracy responsibility, access disparities

## Results: key domains of AI application

The literature reveals that AI technologies have been deployed across multiple domains in humanitarian healthcare, including disaster response, infectious disease surveillance, mental health support, robotic-assisted care, supply chain logistics, and crisis communication. These applications are unified by their aim to enhance speed, precision, and equity in healthcare delivery during emergencies. In the following sections, we present notable examples illustrating how AI is being implemented across each of these domains, with emphasis on real-world impact and challenges.

### AI in disaster response

One striking example of AI’s role in crisis management occurred during the Los Angeles wildfires (Rubin, 2025). As flames spread across communities, AI-driven predictive modeling played a crucial role in assessing fire trajectories, optimizing evacuation routes, and ensuring that medical teams were deployed effectively (Giannakidou et al., 2024). AI-powered drones provided real-time imaging and data analysis, allowing responders to identify hotspots and prioritize areas that required immediate intervention (Buchelt et al., 2024). In hospitals, AI-assisted triage systems helped medical professionals manage the influx of burn victims and respiratory cases, ensuring that resources were allocated where they were needed most (Tshimula et al., 2024b; Timbie et al., 2013; Maleki Varnosfaderani and Forouzanfar, 2024). These technologies not only enhanced the speed of response but also improved outcomes for those affected by the disaster.

Similarly, AI has played a critical role in hurricane relief efforts. During Hurricane Harvey in 2017, AI-driven chatbots and automated response systems helped coordinate emergency relief, directing affected individuals to shelters, food distribution centers, and medical aid stations. Organizations such as the Red Cross and FEMA have increasingly relied on AI-based mapping and analytics tools to assess damage, identify communities in need, and deploy aid strategically. These systems reduce response time, ensuring that assistance reaches those who need it most (Huang et al., 2022).

AI has also been instrumental in earthquake response efforts (Pwavodi et al., 2024). After the 2023 Türkiye-Syria earthquake, AI-powered seismic analysis tools helped predict aftershocks, allowing rescue teams to plan safer operations (Zhao et al., 2023). AI-assisted robots were deployed to navigate rubble and locate survivors in collapsed buildings, significantly improving search and rescue operations (Lokmic-Tomkins et al., 2023). Additionally, AI-driven platforms were used to analyze social media data and satellite imagery, identifying distressed areas that had not yet received aid, ensuring a more efficient allocation of resources (Bari et al., 2023; Mitrović, 2025; Rane et al., 2024).

### AI in addressing the health needs of displaced populations

Beyond natural disasters, AI is proving invaluable in addressing the health needs of displaced populations (Afolabi et al., 2023). Refugee camps, often overwhelmed by the demand for medical care, are now benefiting from AI-driven telemedicine solutions (Haykal et al., 2024; Haykal et al., 2023). Machine learning algorithms analyze patient data to identify disease patterns, enabling healthcare providers to predict and prevent outbreaks before they spiral out of control (Cantini et al., 2025). For instance, AI models have been used to track and contain cholera outbreaks in refugee settlements by analyzing water quality data and sanitation infrastructure (Ahmad Amshi et al., 2024; Ibrahim et al., 2025; Tshimula et al., 2024a; Shannon et al., 2019).

AI chatbots are also being used to bridge language barriers, ensuring that displaced individuals receive accurate health information in their native languages. In Jordan’s Zaatari refugee camp, AI-driven health assistants help Syrian refugees navigate available healthcare services, providing guidance on vaccinations, maternal health, and chronic disease management (Abdulhaq et al., 2024). One notable initiative is the Children Immunization App (CIMA), designed to improve vaccination follow-up among refugee populations. A non-randomized controlled trial conducted between March and December 2019 assessed the app’s effectiveness in the Zaatari camp. The study involved 936 infants, with 471 in the intervention group using the app. Results indicated that 26% of mothers in the intervention group returned for follow-up vaccinations within one week, compared to 22% in the control group. Additionally, the intervention group exhibited a 19% relative risk reduction in loss to follow-up, highlighting the app’s potential to enhance vaccination adherence among refugee children (El-Halabi et al., 2022). Moreover, wearable AI-powered health monitors are being distributed to track vital signs in vulnerable populations, allowing medical teams to intervene at early stages of disease progression. One such instance is UNICEF piloting AI-driven mobile application (MERON) to detect malnutrition in children through image analysis, significantly improving early detection and intervention.

### AI in providing psychosocial support for displaced individuals and disaster survivors

Artificial Intelligence is playing a crucial role in providing mental health support for displaced individuals and those affected by natural hazards. AI-powered counseling bots, such as those developed by Wysa and Woebot, offer psychological assistance to refugees and disaster survivors suffering from trauma, stress, and anxiety (Durden et al., 2023). These chatbots utilize artificial intelligence to provide support through techniques such as cognitive behavioral therapy and mindfulness exercises. They offer immediate assistance in multiple languages, alleviating the burden on overextended healthcare workers and making mental health care more accessible to those in need.

The psychological impact of disasters, including anxiety, post-traumatic stress disorder, and depression, often goes unaddressed due to limited mental health resources in crisis settings (Al Maamari and Elsherbiny, 2025). AI-driven mental health interventions are being integrated into humanitarian responses to offer scalable and accessible support to those affected.

Additionally, AI-driven sentiment analysis tools monitor social media and emergency communication channels to assess the mental health impact of disasters in real time. By analyzing distress signals in text and speech, humanitarian organizations can identify communities in need of mental health interventions and deploy targeted support efforts. AI is also being used to train mental health professionals by simulating crisis counseling scenarios, equipping responders with better tools to manage trauma cases in high-stress environments.

### AI and robotics in emergency healthcare delivery

One of the most promising advancements in AI-driven humanitarian healthcare is the use of robotics to assist in emergency response and medical care delivery (Lokmic-Tomkins et al., 2023). Lessons learned from natural disasters have shown that digital health technologies, including AI-powered robots, can improve healthcare quality and accessibility during crises (Lokmic-Tomkins et al., 2023).

AI-driven robotic systems have been deployed to assist in medical care in hazardous environments where human responders face significant risks. During the COVID-19 pandemic, robotic nurses were used in hospitals to conduct routine patient monitoring, reducing exposure risks for healthcare workers (Sarker et al., 2021; Ashique et al., 2024; Yang et al., 2020). Similarly, in disaster zones, autonomous robots equipped with AI-driven diagnostics can perform basic medical assessments, deliver first-aid supplies, and even administer medications to injured individuals who are trapped or unable to reach a healthcare facility (Bari et al., 2023; Zachariae et al., 2024).

In earthquake-affected regions, search-and-rescue robots use AI-powered sensors to navigate through debris, detect human life, and transmit real-time data to emergency responders (Li et al., 2023). These robots significantly improve the speed and efficiency of rescue operations by accessing hard-to-reach areas that would be dangerous for human teams (Marmaglio et al., 2023).

Furthermore, robotic prosthetics and exoskeletons, integrated with AI, are providing rehabilitation support to disaster survivors who have suffered limb injuries. These robotic systems enhance mobility, accelerate recovery, and offer long-term support for individuals affected by trauma in conflict zones and disaster-stricken areas (Salgado-Gomes-Sagaz et al., 2024; Banyai and Brișan, 2024; Vélez-Guerrero et al., 2021; Samarakoon et al., 2025).

The integration of AI and robotics in humanitarian healthcare is an evolving field, with ongoing research focused on enhancing the autonomy, precision, and effectiveness of robotic medical assistance. As digital health technologies continue to advance, AI-powered robots are expected to play an even greater role in disaster response, providing life-saving medical interventions and improving healthcare accessibility in emergency settings.

### AI for crisis communication and language processing

Language barriers often hinder the effectiveness of humanitarian aid, especially in multicultural refugee camps and disaster-stricken regions. AI-driven natural language processing (NLP) tools are transforming crisis communication by enabling real-time translations of medical instructions, public health advisories, and emergency alerts into multiple languages (Rocca et al., 2023; Kubau and Utulu, 2025). Tools such as Google’s BERT and OpenAI’s models can analyze vast amounts of speech and text data, ensuring that vital health information reaches diverse populations in their native tongues, thereby improving comprehension and compliance with health guidelines (Zhang et al., 2025; Bui et al., 2025).

### AI in disease prediction beyond pandemics

While AI has been instrumental in pandemic response, its predictive capabilities extend to other diseases as well. AI-powered epidemiological models are being used to track and predict the spread of infectious diseases such as malaria, tuberculosis, and dengue fever (Zhao et al., 2024; Gawande et al., 2025; Malik et al., 2020). By analyzing climate data, human migration patterns, and socioeconomic factors, AI can provide early warnings to healthcare organizations, allowing them to allocate resources efficiently and implement preventive measures (Abernethy et al., 2022; Kaur et al., 2021; Ogbaga, 2023). To illustrate, IBM’s Watson Health has been utilized in malaria control efforts by companies like ZzappMalaria, which employs AI to predict high-risk zones and optimize intervention strategies. ZzappMalaria’s AI-powered app analyzes satellite imagery and environmental data to identify potential mosquito breeding sites, enabling targeted larviciding operations. This approach has demonstrated increased coverage of water bodies, reduced work time, and enhanced effectiveness of malaria elimination campaigns.

### AI for supply chain and resource allocation

AI is also transforming humanitarian logistics by optimizing the distribution of medical supplies, food, and relief aid. Predictive analytics models help aid organizations like the World Food Programme and Médecins Sans Frontières (Doctors Without Borders) streamline supply chains, reduce waste, and ensure that resources reach those in need without unnecessary delays. AI-powered drones have also been deployed to deliver vaccines and emergency medical supplies to remote or inaccessible areas, further improving crisis response efforts.

### AI and blockchain for healthcare security

Ensuring the security and integrity of medical records is critical in humanitarian healthcare. AI, combined with blockchain technology, is being used to create secure and tamper-proof digital identities for displaced individuals, allowing them to access consistent healthcare regardless of their location (Corte-Real et al., 2022; Merhej et al., 2024). This approach minimizes the risk of losing critical medical history during forced migrations and ensures that healthcare providers have accurate records when administering treatments. Blockchain-backed AI solutions are already being piloted in refugee camps to streamline patient tracking and medical data sharing while maintaining privacy and security (Abraha, 2025).

### AI and climate-related disaster preparedness

AI is playing an increasingly important role in climate-related disaster response, significantly enhancing forecasting, preparedness, and mitigation efforts. Predictive AI models analyze vast amounts of environmental and meteorological data, such as satellite imagery, atmospheric readings, ocean currents, and historical weather patterns, to identify early signs of extreme weather events (Camps-Valls et al., 2025). By leveraging machine learning and deep learning algorithms, these systems can provide highly accurate forecasts for droughts, floods, heatwaves, and hurricanes, enabling early intervention strategies to minimize damage and loss of life.

One of the most impactful applications of AI in disaster preparedness is its ability to provide early warning systems. In Africa, AI-driven models are being used to predict locust swarms that threaten food security for millions (Enns et al., 2022). These models analyze wind patterns, soil moisture levels, and vegetation growth to forecast swarm movements, allowing governments and farmers to take preventive measures such as targeted pesticide use or alternative agricultural strategies (Javaid et al., 2023). Beyond locust swarms, AI-powered early warning systems are also revolutionizing flood prediction and management. In countries prone to severe monsoons or flash floods, AI models analyze real-time rainfall data, river levels, and terrain maps to predict where flooding is likely to occur (Mosavi et al., 2018; Koutsovili et al., 2023). Notably, Google’s Flood Forecasting Initiative provides hyper-local flood predictions in countries like India and Bangladesh, helping communities prepare before the disaster strikes. In the case of heatwaves, AI models analyze atmospheric pressure changes, land surface temperatures, and historical heat patterns to predict extreme temperature events. This helps public health authorities issue timely advisories, set up cooling centers, and implement protective measures for vulnerable populations, such as the elderly and outdoor workers (Patel et al., 2022; Alvarez et al., 2024).

**Figure 1:**
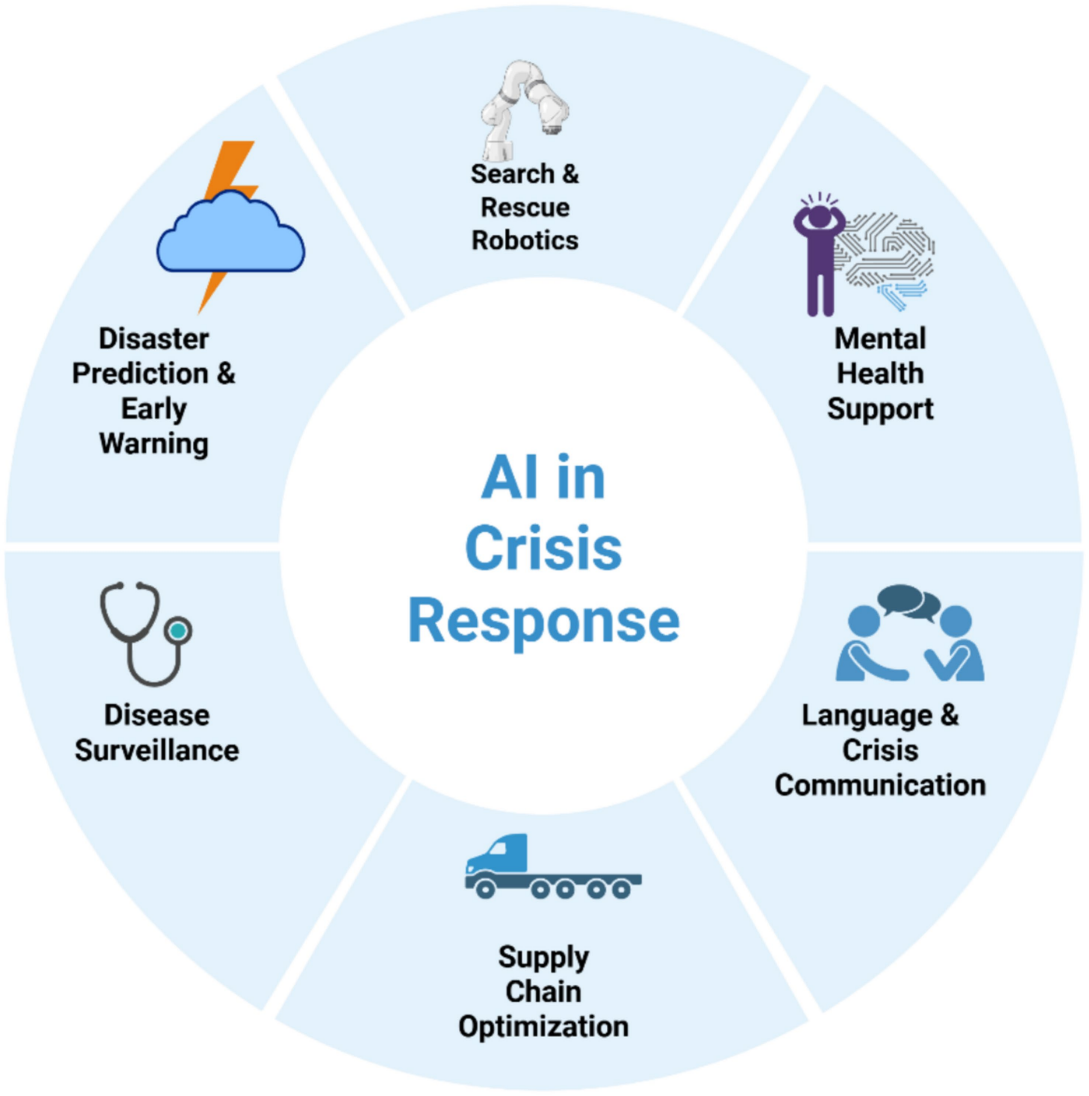
AI-powered humanitarian healthcare in crisis zones.

Integrating AI into climate risk assessments empowers policymakers, humanitarian organizations, and disaster management agencies to develop proactive strategies that mitigate the impact of disasters before they escalate into full-blown humanitarian crises (Barriopedro et al., 2023). As AI technology continues to advance, its role in climate resilience will only strengthen, equipping communities worldwide with the tools needed to confront the growing challenges of extreme weather events with greater preparedness and efficiency (Olawade et al., 2024). Figure 1 illustrates the key domains where AI is actively contributing to crisis response: disaster prediction and early warning, disease surveillance, search and rescue robotics, mental health support, language and crisis communication, and supply chain optimization. These interconnected areas highlight the comprehensive role AI plays across the humanitarian healthcare continuum, from anticipating crises to managing resources and delivering care in real time.

### Ethical concerns

The integration of AI into humanitarian healthcare presents significant ethical challenges that must be addressed to ensure responsible and equitable implementation (Gerke et al., 2020). One of the foremost concerns is bias in AI algorithms, which can lead to disparities in medical decision-making and resource allocation, particularly for vulnerable populations such as refugees and disaster survivors (Farhud and Zokaei, 2021). Ensuring fairness requires diverse and representative training datasets, continuous monitoring, and transparency in AI-driven recommendations. Additionally, data privacy and security are critical, as AI relies on vast amounts of sensitive health information, often collected from individuals in crisis situations without clear mechanisms for informed consent. Protecting this data through encryption, anonymization, and adherence to regulatory frameworks like GDPR is essential (Khalid et al., 2023). Another ethical challenge lies in accountability, when AI-driven decisions impact patient care, it remains unclear whether responsibility lies with the developers, healthcare providers, or humanitarian organizations. Transparency in AI models and human oversight in critical medical decisions are necessary to mitigate risks (Mennella et al., 2024). Moreover, while AI-powered solutions have the potential to improve access to healthcare, disparities in digital infrastructure and internet connectivity may exclude certain populations, exacerbating existing inequalities (Chen et al., 2023; Badr et al., 2024). The deployment of AI in crisis settings should be accompanied by efforts to bridge the digital divide, ensuring that underserved communities benefit from these advancements (Wong et al., 2025). Finally, the governance of AI in humanitarian contexts must align with ethical principles of neutrality, impartiality, and humanity, with collaborative oversight from governments, non-governmental organizations, and healthcare professionals to ensure AI-driven interventions prioritize human rights, dignity, and well-being. Table 1 provides an overview of key AI applications currently shaping the humanitarian healthcare landscape, each representing a promising step toward more responsive, data-driven, and inclusive care during crises. Ethical considerations are implicit in several of these tools: for example, mental health chatbots such as Woebot and Wysa raise concerns about data privacy and the limits of algorithmic empathy; blockchain-backed medical ID tools address challenges of secure data management; and NLP-driven health communication platforms must balance accessibility with potential bias in language interpretation. These examples underscore the need for ethical oversight in both the design and deployment phases of AI solutions in humanitarian settings.

## Conclusion

The integration of AI into humanitarian healthcare is reshaping crisis response, medical interventions, and resource distribution, offering transformative solutions that enhance the efficiency and effectiveness of aid delivery. From disaster prediction and disease surveillance to mental health support and supply chain optimization, AI-driven technologies are addressing some of the most pressing challenges faced in emergency settings.

This review highlights a growing number of real-world applications across these domains, showing how AI can support both acute medical response and longer-term system resilience. However, to ensure these technologies are used responsibly and equitably, ethical challenges, such as algorithmic bias, data privacy, and unequal access to digital infrastructure, must be proactively addressed.

A collaborative approach is essential. By uniting governments, NGOs, academic institutions, and technology providers, we can ensure that AI systems are designed and deployed with inclusivity, transparency, and ethical rigor at their core. With responsible innovation and global cooperation, AI has the potential to become a cornerstone of more adaptive, efficient, and human-centered humanitarian healthcare systems.

## Data Availability

The original contributions presented in the study are included in the article, further inquiries can be directed to the corresponding author/s.
